# Predicting lncRNA–protein interactions through deep learning framework employing multiple features and random forest algorithm

**DOI:** 10.1186/s12859-024-05727-4

**Published:** 2024-03-12

**Authors:** Ying Liang, XingRui Yin, YangSen Zhang, You Guo, YingLong Wang

**Affiliations:** 1 College of Computer and Information Engineering, Jiangxi Agricultural University, Zhimin Avenue, Nanchang, China; 2 First Affiliated Hospital, Gannan Medical University, Medical College Road, Ganzhou, China

**Keywords:** LncRNA–protein interactions, Multiple features, Random forest algorithm, Features fusion

## Abstract

RNA-protein interaction (RPI) is crucial to the life processes of diverse organisms. Various researchers have identified RPI through long-term and high-cost biological experiments. Although numerous machine learning and deep learning-based methods for predicting RPI currently exist, their robustness and generalizability have significant room for improvement. This study proposes LPI-MFF, an RPI prediction model based on multi-source information fusion, to address these issues. The LPI-MFF employed protein–protein interactions features, sequence features, secondary structure features, and physical and chemical properties as the information sources with the corresponding coding scheme, followed by the random forest algorithm for feature screening. Finally, all information was combined and a classification method based on convolutional neural networks is used. The experimental results of fivefold cross-validation demonstrated that the accuracy of LPI-MFF on RPI1807 and NPInter was 97.60% and 97.67%, respectively. In addition, the accuracy rate on the independent test set RPI1168 was 84.9%, and the accuracy rate on the Mus musculus dataset was 90.91%. Accordingly, LPI-MFF demonstrated greater robustness and generalization than other prevalent RPI prediction methods.

## Introduction

Non-coding RNAs (ncRNAs) are the vast majority of RNAs in the sequence of the human genome that do not code for proteins. Short non-coding RNAs (sncRNAs) are non-coding RNAs with less than 200 nucleotides, while long non-coding RNAs (lncRNAs) have more than 200 nucleotides [1]. Recent research has demonstrated that lncRNAs interact with RNA-binding proteins and play essential roles in biological processes [2], including transcription, epigenetic regulation, regulation of cell differentiation, and cell cycle function [3]. Moreover, lncRNAs are intricately associated with high-risk diseases, including cancer. The interaction between lncRNAs and RNA-binding proteins plays a crucial role in the physiological functions of organisms. Consequently, the precise prediction of RPI is indispensable for comprehending the role of lncRNAs in physiological processes.

Previously, some RPI experiments were prohibitively expensive and time-consuming, for example, BIACORE, the main components are optical system, liquid sampling system and sensor chip. It based on surface plasmon resonance (SPR), provides both equilibrium and kinetic information about intermolecular interactions, could obtain detailed insight into the interaction between RNA and proteins carrying RNA recognition motif (RRM) domains [4]. In the past decade, deep learning-based model training has gained attention from data scientists due to its effectiveness in handling large data sets, its high proficiency in training, and its ability to represent features without human intervention automatically [5]. In recent years, numerous RPI prediction methods have been proposed, the majority of which predict RPI using machine learning or deep learning. For instance, Li et al., presented the Capsule-LPI model [6] based on sequence, motif information, physicochemical properties, and secondary structure, and then predicted RPI via a capsule network. Based on sequence features and secondary structure features, Peng et al., presented the RPITER [7] and predicted RPI using convolutional neural networks (CNN) [8] and stacked auto encoder (SAE) [9]. In addition, Yu et al., presented a model known as RPI-MDLStack [10] that predicted RPI interactions based on sequence properties of RNAs and proteins, followed by feature selection by the least absolute shrinkage and selection operator (LASSO) [11] approach and integration of multilayer perceptron (MLP) [12], support vector machine (SVM), RF and gate recurrent unit (GRU). Moreover, Wang et al., presented a model named EDLMFC [13] that predicted RPI using CNN and bilateral-long short term memory (BLSTM) based on sequence and secondary structure. Furthermore, Zhou et al., presented a model named PRPI-SC [14] that predicted RPI using CNN and stacked denoised autoencoder (SDAE) based on sequence and secondary structure. In addition, Huang et al., introduced a model known as LGFC-CNN [15], which is based on the sequence information of RNA and protein, then obtained the secondary structure features through Fourier transform [16], and finally predicted RPI using CNN.

Currently, there is a growing array of RPI prediction methods that exhibit enhanced precision. The steps of RPI prediction based on computational methods include feature extraction, feature fusion, feature selection and classification. While some prior methods only encode the sequence information of RNA and proteins, our model encodes more information, including RNA and protein sequence information, secondary structure information, physical and chemical property information, and protein-protein interaction information. This allows for a fuller capture of the direct RNA-protein interaction. Feature selection will be used to acquire superior features and enhance the model’s generalization capacity since feature fusion can prolong the training duration of features and thereby reduce the model’s predictive capacity. Prior methods that fail to incorporate a feature selection process will result in diminished accuracy of the model’s RPI forecast. Our model utilizes the RF feature selection algorithm to perform feature selection on the feature-encoded data and selects high-quality features to enhance the model’s prediction ability. The advent of deep learning has significantly enhanced the efficacy of RPI forecasting and diminished its predictive expenditure. There are many widely used deep learning techniques, including Graph Convolutional Networks(GCN) [17], Convolutional Neural Networks(CNN), and Long Short-Term Memory(LSTM) [18]. Our model utilizes CNN for RPI prediction and achieves excellent results. Due to the link between lncRNAs and high-risk diseases like cancer, the interaction between lncRNA and RNA-binding protein is crucial for organismal physiological functions. Therefore, the future direction of this model is to enhance our knowledge of the biological functions of lncRNA. Consequently, our study proposes a novel RPI prediction method dubbed LPI-MFF, which combines multiple features, such as protein-protein interaction (PPI) features, sequence features, secondary structure features, and physicochemical properties features, and encodes characteristics using various methods. Subsequently, the RF feature selection algorithm was used to filter out and combine important feature vectors. Finally, a CNN-based deep learning model was used to predict the combined feature information. Thus, a five-fold cross-validation strategy [19] was used in this study to assess the performance of LPI-MFF. Accordingly, in the two datasets (RPI1807 and NPInter), the average ACC of LPI-MFF reached 97.63%, and the values of other evaluation metrics were also higher than those of the majority of RPI prediction methods. The following are the primary contributions of LPI-MFF:

- PPI feature, sequence feature, secondary structure feature, and physical and chemical property feature were used as the four information sources for prediction, improved k-mer (IK) was used to extract sequence information of lncRNA, and the improved conjoint triad (ICT) was used to extract sequence information of protein. Subsequently, Fourier transform was used to extract secondary structure information of lncRNA and protein, and the mapping method of the String database was used to obtain the PPI information. Furthermore, PC-PseAAC and DACC of the Pse-in-One tool were used to extract the physicochemical properties information of lncRNA and protein, respectively. The growth of multi-source information expansion was able to improve the prediction accuracy to some degree, as demonstrated by our subsequent experiments.
- The RF feature selection algorithm was used to screen the four feature vectors based on the Gini index to obtain the optimal. Accordingly, the model’s training pace could be accelerated and the interference of invalid features on the model could be reduced, thereby increasing the robustness of the model, making it easier for the classifier to process the information.
- After features fusion, a CNN-based deep learning method was used to predict the feature vectors. This feature reuse method could fully extract the information contained in the effective features after feature filtering, thereby enhancing the accuracy of the model.

## Materials and methods

### Datasets

In deep learning, the collection or development of a valid benchmark dataset is important, to train a computational model [20]. The selection of a suitable benchmark has a high impact on the performance rates of a model [21]. Our investigation made use of two datasets from the PDB database [22] and NPInter v2.0 database [23]: RPI1807 [24] and NPInter [25]. RPI1807 was determined by measuring the distance between RNA and protein atoms, it consists of 1807 RPI pairs and 1436 non-RPI pairs, which are represented by 1078 RNAs and 3131 proteins. NPInter collects the records of lncRNA and protein interaction experiments in the NPInter v2.0 database. This database contained 10,412 RPI pairs and 0 non-RPI pair, which were represented by 4636 RNAs and 449 proteins. Due to the absence of non-RPI pairs in NPInter, this results in a lack of negative samples in the training model, so the same number of non-RPI pairs as RPI pairs were selected randomly from the proteins and RNAs that excluded the RPI pair.

Simultaneously, an independent dataset known as RPI1168 was introduced to validate the model’s generalization performance. The dataset consisted of 1168 RPI and 1168 non-RPI pairs. The positive pairs were obtained from RPI2241 [26]. Additionally, we removed the RPI pairs with RNA sequences (<200nt) in RPI2241 and screened them to finally obtain 1168 RPI pairs and 0 non-RPI pair, which were represented by 421 RNAs and 1035 proteins, since RPI2241 had no non-RPI pairs like NPInter, this results in a lack of negative samples in the training model, the same number of non-RPI pairs as RPI pairs were randomly selected from proteins and RNAs that excluded the RPI pairs. The dataset used for this study is described in Table 1.

**Table 1:** Describe the datasets that were used in this study

Datasets	RPI pairs	Non-RPI pairs	RNAs	Protein
RPI1807	1807	1436	1078	3131
NPInter	10412	10412	4636	449
RPI1168	1168	1168	421	1035

### Multimodal features coding

**Fig. 1:**
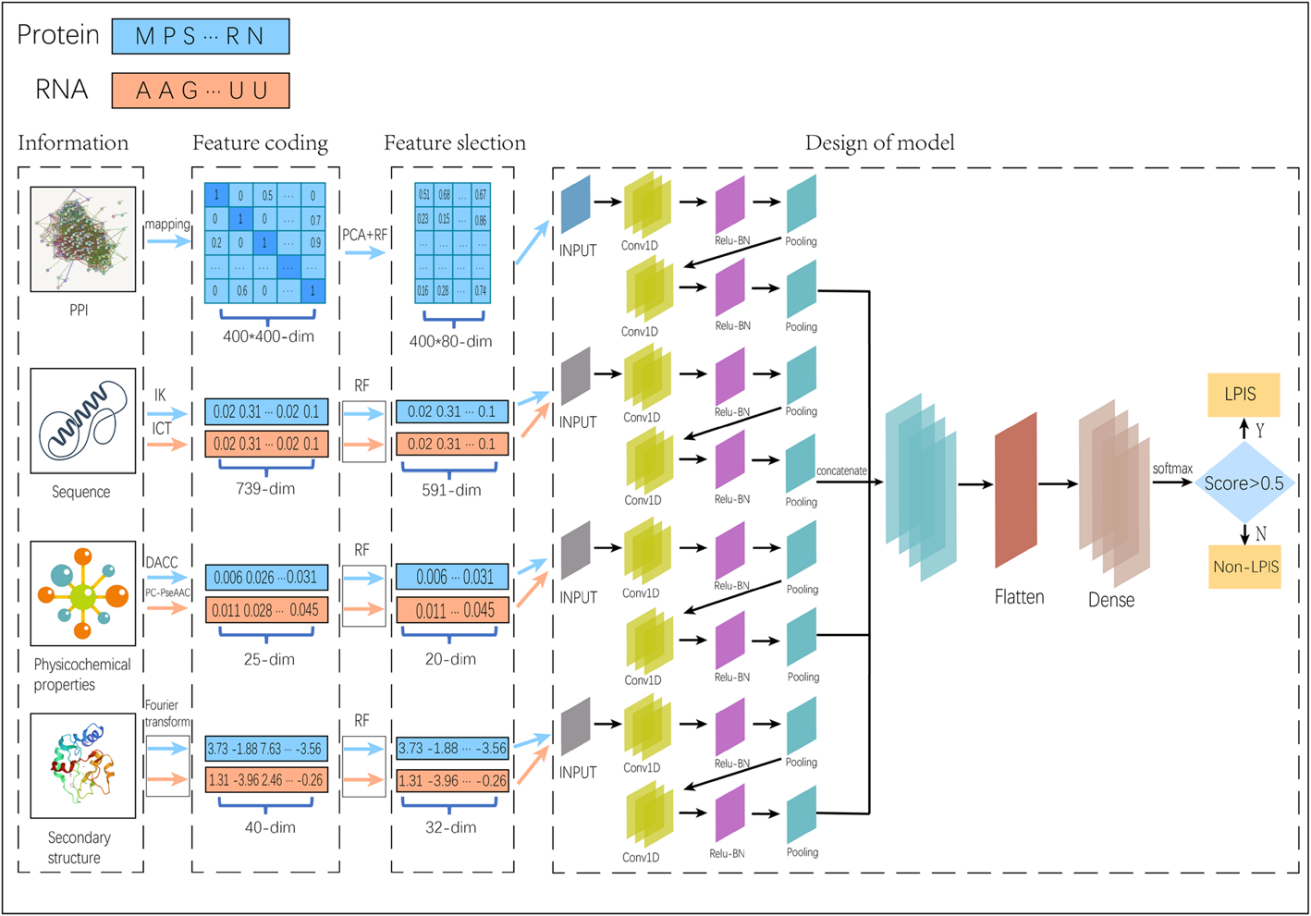
The flowchart of LPI-MFF

This study predicts RPI using multiple types of information, including PPI information, sequence information, secondary structure information, and physicochemical properties information, to predict RPI. Thus, for various types of data, corresponding feature encoding techniques were used. The RPI prediction method LPI-MFF is depicted in Fig. 1. The pipeline of this flowchart is as follows: (1) Multi-source information extracting. Utilizing PPI feature, sequence feature, secondary structure feature, and physical and chemical property feature as information sources. (2) Feature encoding. Use mapping method from the String database to encode the features of PPI information, IK to encode the features of the RNA sequence information, ICT to encode the features of the protein sequence information, DACC to encode the features of the physicochemical properties of RNA and PC-PseAAC to encode the features of the physicochemical properties of protein, Fourier transform to encode the features of the secondary structure information. (3) Feature selection. Use the RF feature selection algorithm to screen the feature vectors to reduce the interference of invalid feature vectors on the model. (4) Model construction. The deep learning method based on CNN is used to concatenate all the information together for feature fusion, further improve the accuracy of the model. (5) Model evaluation. Five-fold cross-validation is performed on RPI1807, NPInter, and RPI1168, respectively, to identify the model’s performance indicators, and compare LPI-MFF to other RPI prediction methods to demonstrate its superiority. The subsequent four subsections introduce the information and the corresponding encoding methods.

#### PPI features

The PPI data used in our study can be accessed from the STRING database [27]. The STRING database contains a large amount of biological protein-protein association data, which can be accessed by uploading the fasta file. The PPI information is in the form of “2AKE-A” id, consequently, LPI-MFF used the mapping method from the String database to map. In addition, the PPI information was in the form of a confusion matrix [28]. Each value in the confusion matrix represented the interaction strength between two proteins, and the matrix’s dimensions were 400 by 400. Additionally, the PPI information was expressed as a 400–400 matrix:

1 $$\begin{aligned}&PPI=\left[\begin{array}{lllll} 1.0_{1,1} &{} 0.0_{1,2} &{} 0.5_{1,3} &{} \ldots &{} 0.0_{1,20} \\ 0.0_{2,1} &{} 1.0_{2,2} &{} 0.0_{2,3} &{} \ldots &{} 0.7_{2,20} \\ 0.2_{3,1} &{} 0.0_{3,2} &{} 1.0_{3,3} &{} \ldots &{} 0.9_{3,20} \\ \ldots &{} \ldots &{} \ldots &{} \ldots &{} \ldots \\ 0.0_{20,1} &{} 0.6_{20,2} &{} 0.0_{20,3} &{} \ldots &{} 1.0_{20,20} \end{array}\right]&\end{aligned}$$

High-dimensional feature vectors are affected by the curse of dimensionality, therefore, LPI-MFF employed the Principal Component Analysis (PCA) [29] method to reduce the dimensionality of the confusion matrix to 100 dimensions for each eigenvector. Accordingly, we used the 100-dimensional “0” as PPI feature data for proteins whose PPI cannot be accessed through the String database. Finally, a 400*100-dimensional eigenvector was used to represent the PPI information.

#### Sequence features

LPI-MFF also employed feature encoding methods, IK and ICT, for RNA and protein sequences, respectively, which transformed each information type into a fixed-length feature vector. RNA sequences are composed of four distinct classes of nucleotides: A, U, C, and G. LPI-MFF used the IK to encode RNA sequences from 1-mer to 4-mer, which means that in addition to calculating the frequency information of 4-mer, we also calculate the frequency information of 1-mer, 2-mer, and 3-mer, resulting in the acquisition of a 340($4^{1}$ +$4^{2}$+$4^{3}$+$4^{4}$)-dimensional feature vector representing RNA sequence information. The RNA sequence data was encoded as:

2 $$\begin{aligned}&R_{IK} =\left[ {\textstyle \sum _{i=1}^{4}} f_{1} ,...,f_{4^{i} } \right]&\end{aligned}$$

where, $f_{n}$ represents the frequency of each nucleotide combination, and i represents the number of nucleotides in each nucleotide combination. Accordingly, this method superimposed the feature information of 1-mer, 2-mer, 3-mer and 4-mer. Protein sequences are typically composed of twenty different amino acids. Using the volume characteristics of amino acids and side chains, we categorized them into seven distinct groups: {A, G, V}, {I, L, F, P}, {Y, M, T, S}, {H, N, Q, W}, {R, K}, {D, E}, and {C}. In our study, the ICT coding method of protein sequence changed from 1-mer to 3-mer, which means that in addition to calculating the frequency information of 3-mer, we also calculated the frequency information of 1-mer and 2-mer, allowing for the acquisition of a 399($7^{1}$ +$7^{2}$+$7^{3}$)-dimensional feature vector representing protein information. The information on the protein sequence is expressed as:

3 $$\begin{aligned} P_{ICT} =\left[ {\textstyle \sum _{i=1}^{3}} f_{1} ,f_{2}...,f_{7^{i} } \right] \end{aligned}$$

where,$f_{n}$ represents the frequency of each amino acid combination, and i represents the number of amino acids in each amino acid combination. The method superimposes the feature information of 1-mer, 2-mer and 3-mer.

Thus, combining the 340-dimensional RNA sequence feature vector and the 399-dimensional protein sequence feature vector results in a 739-dimensional sequence feature vector. And we add the pseudocode of the IK and ICT to the Additional file 1.

#### Physicochemical properties features

Using the Pse-in-One 2.0 tool [30], LPI-MFF extracted the physicochemical information of IncRNA and protein. This method is more flexible than Pse-in-One [31] and included 23 new pseudo-component modes and several new feature analysis techniques.

For RNA, LPI-MFF adopts the “DACC” mode in Pse-in-One 2.0, and selects 22 kinds of physicochemical properties, contain content information (Adenine content, GC content, Purine content, Keto content, Cytosine content, Thymine content, Guanine content), dynamic information (Tilt, Twist, Roll, Rise, Shift, Slide), energy information (Stacking energy, Entropy, Entropy 1, Enthalpy, Enthalpy 1, Free energy, Free energy 1), Characteristics (Hydrophilicity, Hydrophilicity 1). For proteins, LPI-MFF used the “PC-PseAAC” mode in Pse-in-One 2.0 and selects hydrophobicity, hydrophilicity, and mass as physicochemical properties. The selection of these physicochemical properties was based on their effectiveness in previous RPI prediction methods. Since the feature vector representing RNA physicochemical properties had 22 dimensions and the feature vector representing protein physicochemical properties had 3 dimensions, combining the two feature vectors yielded physicochemical properties feature vectors with 25 dimensions.

#### Secondary structure features

The secondary structure of RNA and proteins usually arises from helical or folded sequences. Just like sequence data, secondary structure information cannot be directly utilized as input for the prediction model. Therefore, it is necessary to convert the string representation of secondary structure information into digital form, and the length problem of the secondary structure features must be resolved.

LPI-MFF employed the RNAsubopt method implemented in ViennaRNA Package 2.0 for RNA secondary structure [32]. RNAsubopt can acquire the top n secondary structures with the lowest free energy. Since the value of n has little impact on the prediction result, we set n to 5 for the sake of calculation. In addition, its secondary structure output is a string consisting of “.” and “(“ or ”)”. To ensure that the feature vectors of RNA secondary structure have the same dimension, we replaced “.” with “0” and “(“ or ”)” with “1”, and then combine to obtain a new feature vector. LPI-MFF then applied a Fourier transform to the obtained feature vector and selects the first 20 elements of the Fourier series as the new feature vector. LPI-MFF uses the SSpro method implemented in SSpro/ACCpro 6 [33] for determining the secondary structure of proteins. The method predicts the secondary structure of proteins based on three types of features ($\alpha$-helix, $\beta$-sheet, coil), and its protein secondary structure output consists of strings containing “C”, “E”, and “H”. Protein secondary structure used a similar method to RNA secondary structure to solve the secondary structure length problem. LPI-MFF replaces “C” with “0”, “E” with “1”, and “H” with 2. Subsequently, the resulting feature vectors were Fourier transformed, and the first 20 elements of the Fourier series were selected as the new feature vector. Accordingly, a 20-dimensional feature vector representing the protein secondary structure was acquired. The Fourier transform formula is as follows:

4 $$\begin{aligned} X_{i} =\sqrt{\frac{2}{l} } \sum _{n=0}^{l} X_{n} \cos \left[ \frac{\pi }{l}\left( n+\frac{1}{2} \right) \left( i+\frac{1}{2} \right) \right] ,i=0,1,2,...,19 \end{aligned}$$

where, l represents the length of the feature vector, n represents the number of feature vector values, and i represents the number of items for Fourier transformation. This method converted the first 20 items of the Fourier series into new feature vectors, and the secondary structure information into 20-dimensional feature vectors. Combining the feature vectors representing the secondary structure of RNA and protein, LPI-MFF had a total of 40-dimensional feature vectors representing the secondary structure.

### Feature selection algorithm based on RF

High-dimensional feature vectors can lead to the curse of dimensionality, potentially causing overfitting in predictive models and extended calculation times. In order to prevent issues caused by high dimensions, such as overfitting, LPI-MFF used RF [34] to perform feature selection based on the Gini index, the variable importance measures (VIM) [35] were computed using the Gini index, and the feature vector with the highest VIM is selected for input into the prediction model. The Gini index has the following formula:

5 $$\begin{aligned} GI= {\textstyle \sum _{i=1}^{n}} {\textstyle \sum _{i\ne 1}^{}} P_{ik^{2} } \end{aligned}$$

where n represents the number of categories, $P_{ik}$ represents the category k on the i node. The formula for the VIM is as follows:

6 $$\begin{aligned} VIM=GI_{i} -GI_{r} -GI_{l} \end{aligned}$$

where $GI_{i}$ represents the GI of the node, $GI_{l}$ represents the GI of the left subtree of the node, and $GI_{r}$ represents the GI of the right subtree of the node. When N decision trees are present, the formula for the VIM is represented as follows:

7 $$\begin{aligned} VIM= {\textstyle \sum _{i=1}^{n}} VIM_{i} \end{aligned}$$

where n represents the number of decision trees, and the final value of VIM is the sum of the VIMs of each tree.

Finally, the selected feature vector representing PPI had 320*100 dimensions, the feature vector representing sequence had 591 dimensions, the feature vector representing physicochemical properties had 20 dimensions, and the feature vector representing secondary structure had 32 dimensions. The hyperparameters for RF are in the Additional file 1.

### Design of model

Deep learning models are critical in predicting results, particularly in RPI prediction. Previous RPI prediction algorithms focused solely on RNA and protein sequence and secondary structure features. As an enhancement, LPI-MFF employed not only the sequence and secondary structure features of RNA and protein but also their physicochemical property and PPI features. In this study, a parallel architectural model LPI-MFF with four characteristics was, therefore, developed. In addition to the PPI feature, the remaining three feature vectors integrate the protein feature vector with the RNA feature vector, thereby strengthening the link between the protein and the lncRNA. After feature selection, LPI-MFF inputs the four feature vectors into the three-layer convolutional (Conv) layer with a convolution kernel size of 3*3, and then extracts the corresponding features. Most model features are typically fed into a single activation function, such as sigmoid or tanh. Therefore, we employed the two activation functions of batch normalization (BN) [36] and rectified linear units (ReLU) [37] in order to further accelerate the training speed, improve the classification effect, and prevent overfitting. The feature vector was first input into the ReLU activation function, which prevented the overfitting and enhanced the computing efficiency. Subsequently, the feature vector was input into the BN activation function, and the distribution of each hidden layer was normalized to the standard normal, which prevented the gradient from disappearing and overfitting. Next, the features that had passed through the activation function layer were sent to the pooling layer to eliminate redundant features, reduce the dimension of features, and retain relevant features. The feature was then traversed again to the Conv, activation function, and pooling layers. We used the concatenate method to obtain a feature vector for future predictions, as the recently obtained feature vectors correspond to distinct features. The as-obtained feature vectors were then input into the Flatten layer to make them one-dimensional. The feature vector was then fed into the fully connected (FC) layer of the three layers, where the respective neuron sizes were 16, 8, and 2. Finally, the softmax activation function was used as the final step in binary classification. If the obtained value was greater than 0.5, it was determined to be an RPI pair, otherwise, it is determined to be a Non-LPI pair. In this study, we used backpropagation to minimize the loss function and Adam and stochastic gradient descent (SGD) to train each feature module. The hyperparameters for LPI-MFF are in the Additional file 1.

### Model evaluation

The evaluation index, which compares multiple models using the same evaluation criteria, is a persuasive criterion for comparing our model to other RPI prediction methods. In our study, we evaluated the model using five-fold cross-validation, randomly dividing the samples into five non-repetitive subsets. Four of these subsets were utilized as the training set, while the remaining subset was used as the test set. We repeated this process until each subset had been used as the test set. Finally, the average of the five experimental outcomes was used as the result. The seven distinct evaluation indicators used for the evaluation criterion were: accuracy (ACC), sensitivity (SEN), specificity (SPE), precision (PRE), F1-score (F1), Matthews correlation coefficient (MCC), and area under the curve (AUC) [38]. Their formulas are listed below:

8 $$\begin{aligned} ACC&=\frac{TP+TN}{TP+TN+FP+FN} \end{aligned}$$

9 $$\begin{aligned} SEN&=\frac{TP}{TP+FN} \end{aligned}$$

10 $$\begin{aligned} SPE&=\frac{TN}{TN+FP} \end{aligned}$$

11 $$\begin{aligned} SPE&=\frac{TN}{TN+FP} \end{aligned}$$

12 $$\begin{aligned} F1&=\frac{2\times PRE\times SEN}{PRE+SEN} \end{aligned}$$

13 $$\begin{aligned} MCC&=\frac{TP\times TN-FP\times FN}{\sqrt{(TP+FP) (TP+FN)(TN+FP) (TN+FN)} } \end{aligned}$$

14 $$\begin{aligned} AUC&= \frac{{\textstyle \sum _{i\in P}^{}} r_{i} -\frac{|P|\times (|P|+1)}{2} }{|P|\times |N|} \end{aligned}$$

where TP is the number of correctly projected positive samples, TN is the number of correctly forecasted negative samples, FP is the number of incorrectly predicted positive samples, and FN is the number of incorrectly predicted negative samples. Additionally, P denotes the positive sample set, N denotes the negative sample set. and $\left| P \right|$ denotes the number of elements in the positive sample set. $P^{r_{i} }$ shows the rank position of element i in the entire set (P+N) based on its anticipated score, from small to large. The final result was determined by calculating the outcomes of each evaluation indicator using five-fold cross-validation and averaging the values obtained.

## Result

### The effect of epochs on training and testing results

**Fig. 2:**
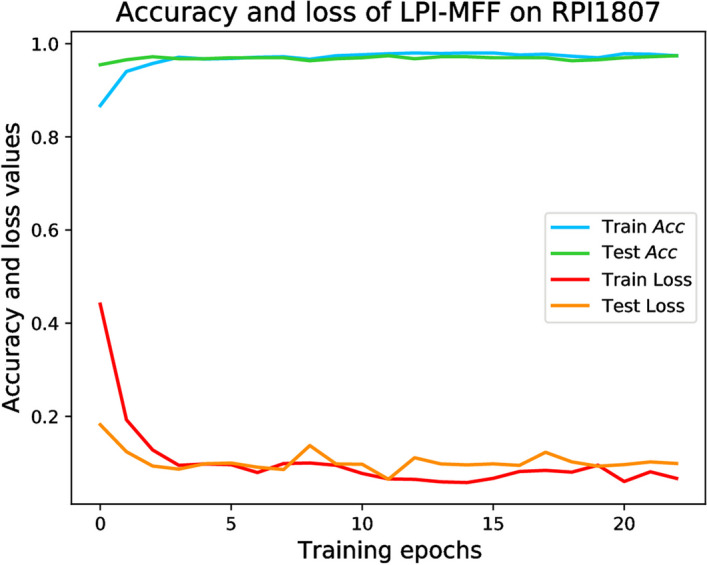
The ROC curve of different feature combinations on RPI1807

Epoch is a significant notion that represents the total number of times the model has gone over the entire dataset during training. Over the course of several epochs, the model progressively adjusts to the training data, enhances its performance, and evaluates its capacity to generalize by examining its verification performance. Properly determining the number of epochs is a crucial hyperparameter in deep learning, which must be fine-tuned based on the particular problem and dataset. We performed tests to assess the influence of epoch size on the accuracy and loss in both the training set and test set using the RPI1807 dataset. The results are depicted in the Fig. 2.

### The effect of various feature combinations on predicted results

In general, the sequence information and secondary structure information of proteins and RNAs are the feature information utilized by the majority of LPI prediction algorithms [39]. LPI-MFF included PPI and physicochemical properties information in addition to RNA and protein sequence and secondary structure information. Therefore, in order to determine whether the four features have an effect on the prediction results and to select the optimal solution, these four features are combined in 11 combinations, and experiments were conducted to evaluate the impact of the different feature combinations on the prediction results. Table 2 depicts the prediction results of various RPI1807 data set combination permutations.

According to Table 2, the feature combination consisting of PPI and physical and chemical qualities had the highest sensitivity, yielding 97.09%. Moreover, the combination of sequence, secondary structure, and PPI information had the highest F1 and MCC values, 95.51% and 0.9769, respectively. Furthermore, the feature combination consisting of sequence, secondary structure, physical and chemical characteristics, and PPI information had the highest ACC, SPE, PPV, and AUC values, which were 97.73%, 99.49%, 99.47%, and 99.55%, respectively. Moreover, PPI information and physicochemical properties information could improve RPI prediction outcomes when using the four features employed in this study. Furthermore, the greater the number of features included in the feature combination, the greater the effect. Accordingly, the feature combination consisting of sequence information, secondary structure information, PPI information, and physicochemical properties information demonstrated the highest four evaluation indicators out of seven evaluation indicators, correspondingly, this feature combination was selected.

**Table 2:** Comparison of prediction results with different feature combinations on RPI1807

Combination of Features	ACC (%)	SEN (%)	SPE (%)	PPV (%)	F1 (%)	MCC	AUC (%)
PPI,PC	94.17	**97.09**	91.25	92.94	89.15	0.9465	99.31
Str, PPI	96.40	94.43	98.37	98.34	92.90	0.9633	99.14
Str, PC	97.47	95.72	99.23	99.21	95.01	0.9743	99.52
Seq, PPI	97.39	95.89	98.88	98.85	94.81	0.9735	99.17
Seq, PC	96.87	96.06	97.68	97.66	93.76	0.9685	99.25
Seq, Str	96.87	95.20	98.54	98.50	93.81	0.9682	99.22
Str, PPI, PC	97.56	95.71	99.40	99.39	95.19	0.9751	99.41
Seq, PPI, PC	94.08	96.23	91.93	93.59	88.93	0.9456	98.85
Seq, Str, PPI	97.60	96.10	99.31	99.29	**95.51**	**0.9769**	99.52
Seq, Str, PC	97.51	95.89	99.14	99.12	95.08	0.9747	99.48
Seq, Str, PPI, PC	**97.73**	95.72	**99.49**	**99.47**	95.27	0.9755	**99.57**

Bold values represent the maximum value of the corresponding evaluation indicator

**Fig. 3:**
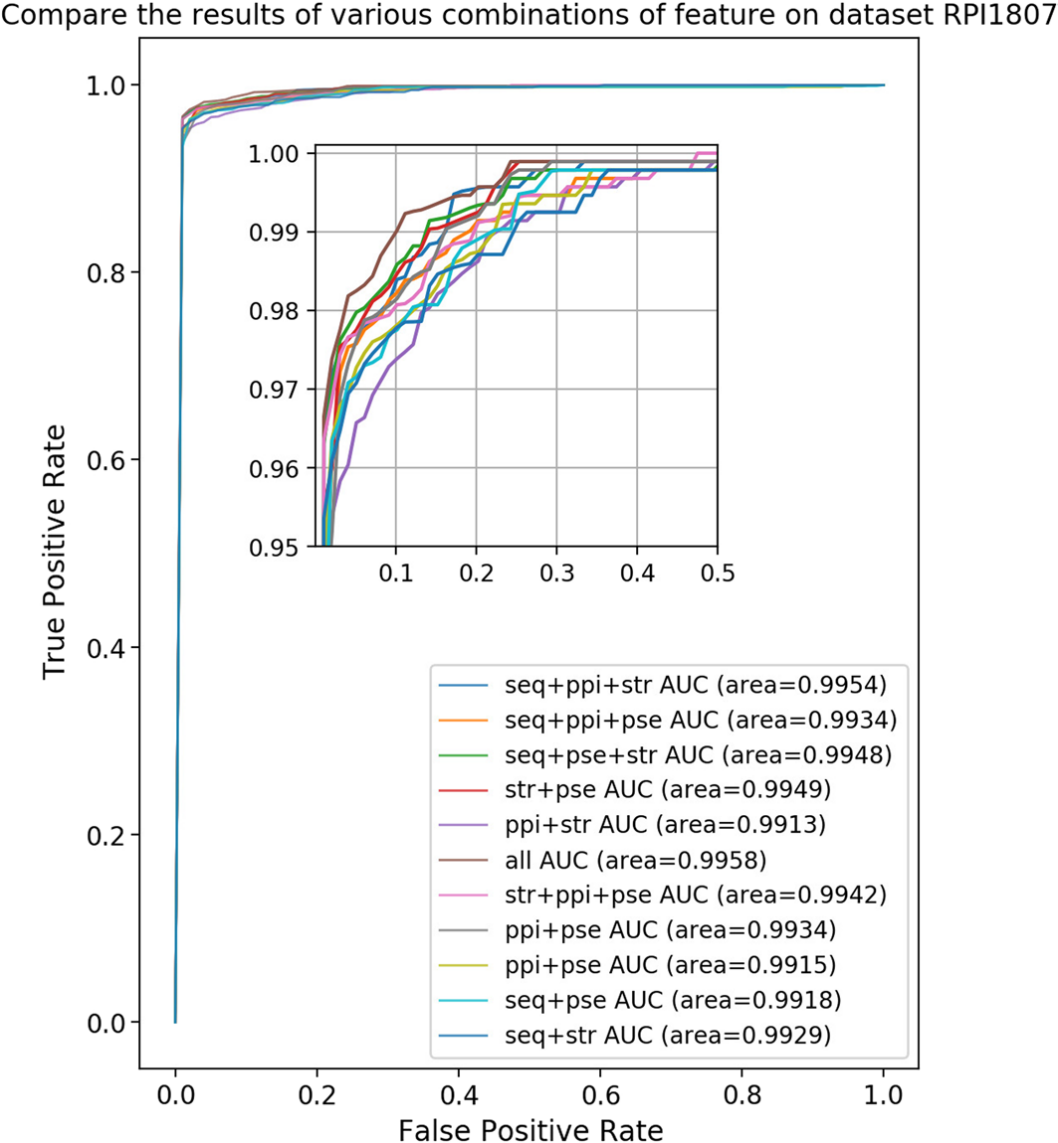
The ROC curve of different feature combinations on RPI1807

In addition, we evaluated the impact of feature combinations on RPI prediction from other perspectives, Fig. 3 depicts the ROC curve with different feature combinations [40].

As shown in Fig. 3, the AUC value for the combination of sequence information, secondary structure information, PPI information, and physicochemical properties information was the highest, reaching 0.9958. The AUC values of the feature combinations containing all feature information are slightly higher than those of the other feature combinations, indicating that this feature combination performs the best among the 11 sets of feature combinations.

### The effect of feature selection algorithms on predicted results

Most RPI prediction models contain feature selection algorithms, which aid in enhancing computational efficiency, removing redundant data, and preventing overfitting. If LPI-MFF did not employ the feature selection algorithm, the dimensions of the last four feature fusions would exceed 1000, which is likely to result in dimensional issues such as overfitting. In order to determine the optimal feature selection approach for this model, LPI-MFF identified five potential methods: spectral embedding (SE) [41], logistic regression (LR) [42], RF, LASSO and elastic net (EN) [43]. To ensure adherence to the concept of a single variable, each feature selection method screened 50% of the feature vectors. Except for the change to the method of feature selection, all other model parameters remain unchanged. Four evaluation indicators (ACC, SEN, SPE, and MCC) were used in Table 3 to demonstrate the effect of feature selection methods on the RPI1807 prediction results.

As listed in Table 3, the SE feature selection algorithm had the highest SPE value, reaching 98.62%. Nonetheless, the RF feature selection algorithm has the highest ACC, SEN, and MCC values, achieving 97.60%, 95.72%, and 0.9755 respectively. Although the SE feature selection algorithm had the highest value of one evaluation indicator, the RF feature selection algorithm demonstrated the highest value of three evaluation indicators, which were significantly higher than the other four feature selection methods, accordingly, the RF feature selection algorithm can be determined to be the best. Thus, for this research model, the RF feature selection algorithm is the optimal choice.

**Table 3:** Comparison of prediction results with different feature selection algorithms on RPI1807

Feature selection algorithms	ACC(%)	SEN(%)	SPE(%)	MCC
SE	96.48	94.34	**98.62**	0.9639
LR	96.31	94.17	98.45	0.9622
RF	**97.60**	**95.72**	98.49	**0.9755**
LASSO	96.95	95.88	98.02	0.9692
EN	91.51	85.93	97.08	0.9098

Bold values represent the maximum value of the corresponding evaluation indicator

**Fig. 4:**
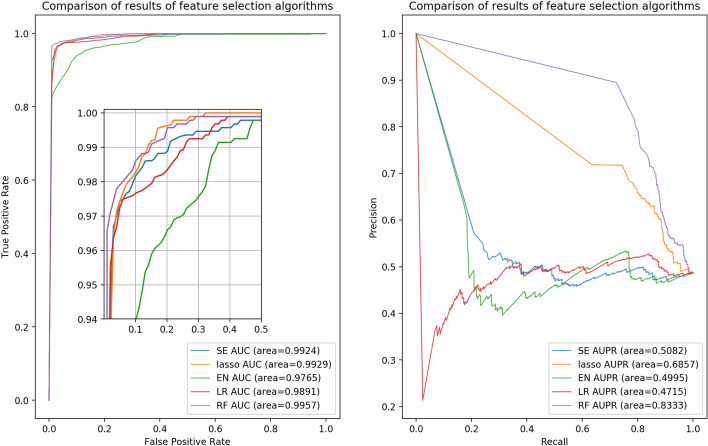
The ROC curves with different feature selection algorithms on RPI1807, and the PR curves with different feature selection algorithms on RPI1807

In addition, we evaluated the influence of feature selection algorithms on RPI prediction from other perspectives, ROC and PR curves for various feature selection algorithms [44] are shown in Fig. 4.

Figure 4 demonstrates that RF had the highest AUC and AUPR values, with values of 0.9957 and 0.8333, respectively. Although the AUC value of RF was slightly higher than the AUC value of the other four feature selection algorithms, the AUPR value of RF was significantly higher than the AUPR value of the other four algorithms. Through the ROC and PR curves, it can be seen that, of the five feature selection algorithms, only RF can filter out the optimal feature vector and achieve the best RPI prediction performance.

### Feature selection ratio’s influence on prediction results

Although the choice of feature selection methodology has an effect on the outcome of the prediction, the proportion of features selected has an equal impact. Thus, screening out feature vectors with the correct proportions can not only prevent dimensional issues such as overfitting, but it can also improve the model’s prediction results to some extent. In order to investigate the effect of the feature selection ratio on the prediction results, LPI-MFF utilized various feature selection ratios [39] and four evaluation indicators (ACC, SEN, SPE, and MCC) to investigate the effect of feature selection ratios on the prediction results. Furthermore, LPI-MFF utilized a distinct feature selection ratio for the sequence feature vector and a unified feature selection ratio [45] for the PPI, physicochemical properties, and secondary structure feature vectors. Table 4 illustrates the impact of the feature selection ratio on the prediction results in the RPI1807 dataset based on four evaluation indicators (ACC, SEN, SPE, and MCC).

As shown in Table 4,When the feature selection ratio of the sequence feature vector was 20% and the feature selection ratios of the PPI, physicochemical properties, and secondary structure were 80%, the SEN value reached a maximum of 95.54%. Additionally, when the feature selection ratio of the sequence feature vector was 80% and the feature selection ratios of the PPI, physicochemical properties, and secondary structure were 80%, the ACC value, SPE value, and MCC value were the highest, reaching 97.5%, 99.83%, and 0.9750, respectively. This suggests that when the feature selection ratio of the sequence feature vector was 80% and the feature selection ratios of the PPI, physicochemical properties, and secondary structure feature vectors were also 80%, the prediction result could be significantly improved, and the optimal feature selection ratio for the model could be determined.

**Table 4:** Comparison of prediction results with different feature selection ratios on RPI1807

Feature selection ratio	ACC (%)	SEN (%)	SPE (%)	MCC
b2s2_re	97.39	95.46	99.23	0.9734
b2s8_re	97.43	**95.54**	99.40	0.9738
b8s2_re	97.17	94.95	99.02	0.9711
b8s8_re	**97.56**	95.29	**99.83**	**0.9750**

Bold values represent the maximum value of the corresponding evaluation indicator

The number after b reflects the proportion of feature screening and retention for sequence, and the number after s represents the proportion of feature screening and retention for PPI, physicochemical properties and secondary structure

### Impact of feature fusion methods on prediction results

Four distinct feature vectors were utilized in LPI-MFF. Thus, in order to maximize the utility of the feature vectors, LPI-MFF utilized feature fusion to combine the four feature vectors in order to improve prediction results and enhance operation efficiency. In order to determine the best feature fusion [46] method for this model, LPI-MFF selected two feature fusion methods, concatenate [47] and stacking, for comparison. Concatenate was used to connect four types of information, and fused the connected information, and it could alleviate dimensional problems such as gradient disappearance, whereas stacking was used to connect four types of information in series, it could effectively combat over-fitting and does not require too much parameter adjustment. Combine the prediction results of the training set and the test set as the new training set and test set respectively. Table 5 illustrates the impact of these two feature fusion strategies on the prediction results for the RPI1807 dataset via four evaluation indicators (ACC, SEN, SPE, and MCC).

As shown in Table 5, ACC, SEN, SPE, and MCC values of the concatenate method were higher than those of the stacking method, reaching 97.60%, 95.72%, 99.49%, and 0.9755, respectively, as shown in Table 5. Since the concatenate method yielded the highest values for all evaluation indicators, the concatenate method is best suited for this model.

**Table 5:** Comparison of prediction results for different feature fusion strategies on RPI1807

Feature fusion algorithms	ACC(%)	SEN(%)	SPE(%)	MCC
Concatenate	**97.60**	**95.72**	**99.49**	**0.9755**
Stacking	96.41	94.31	98.77	0.9631

Bold values represent the maximum value of the corresponding evaluation indicator

### Interpret the model using LIME and SHAP

**Fig. 5:**
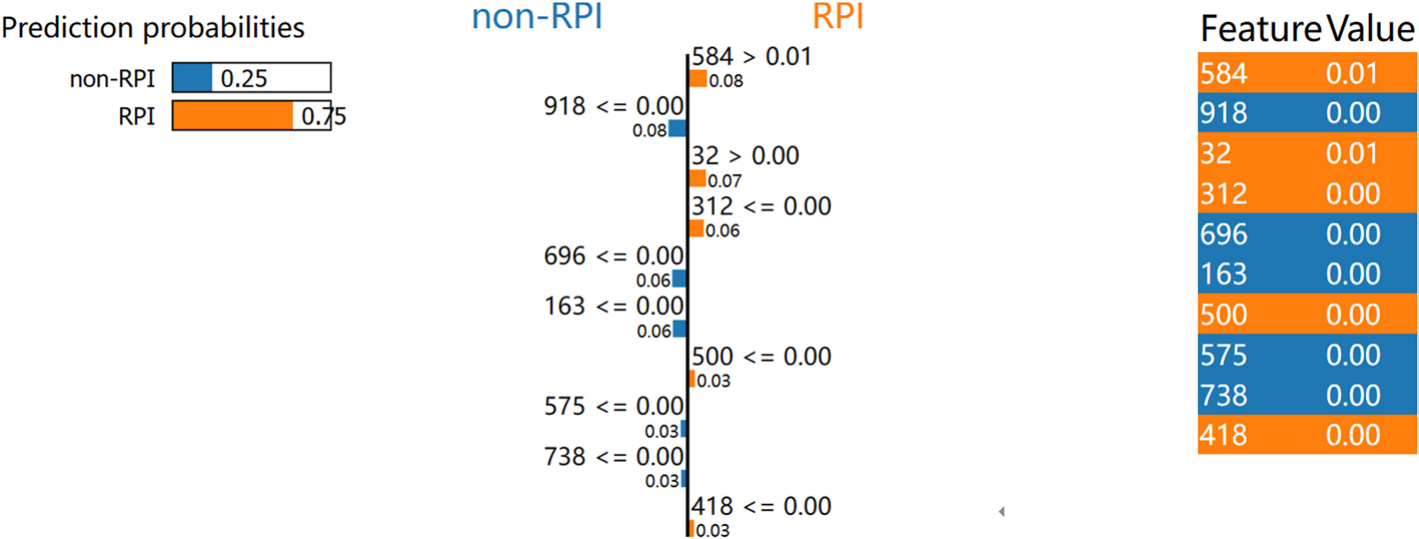
LIME analysis of LPI-MFF

**Fig. 6:**
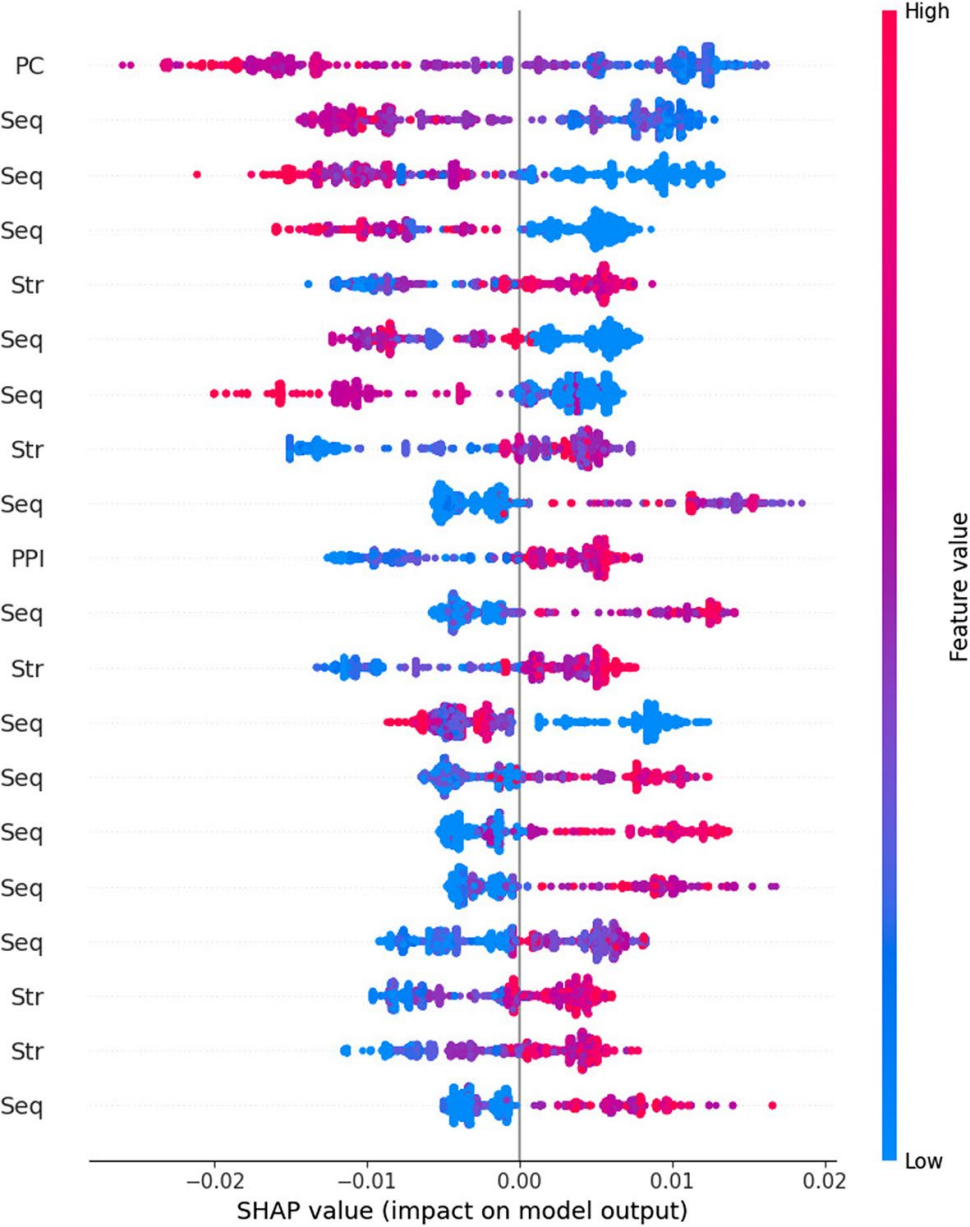
SHAP analysis of LPI-MFF

Biologically relevant feature extraction is not a simple process. Deep learning-based training models are commonly referred to as “black boxes” due to their intricate mechanics. Calculating the contribution of each feature in the model is a challenging task. We employ the Local Interpretable Model-Agnostic Explanation (LIME) and Shapley Additive Explanation Algorithm (SHAP) [48] in our research to provide explanations for LPI-MFF. These methods investigate the contribution of the extracted features by visualizing the high contributory features from the whole feature set using machine learning algorithms [49]. The essence of LIME lies in utilizing the initial input features and model prediction values to elucidate the prediction value of each individual sample by means of the local surrogate model. We randomly selected a feature to perform LIME analysis on it, as shown in the Fig. 5, 25% of the forecasts are for non-RPI, and 75% of the forecasts are for RPI. Our feature dimension is very high and has been normalized, so the value of each feature is very small, and some are in the range of 1.0e$-$4, so 0.00 is displayed. SHAP is an interpretation technique that draws on game theory ideas. SHAP quantifies the influence of individual features by computing the incremental effect of each feature in the model, and thereafter elucidates the functioning of the black-box model. The Shapley Value in SHAP refers to the marginal contribution. As shown in Fig. 6, SHAP analysis shows the top 20 significant features. Every feature is allocated a SHAP value, which indicates the distribution of the SHAP value for that the feature and reflects its effect in the trained model. Where a red dot signifies a higher feature value and a blue dot indicates a lower one. These colors display the direction of the features based on their predicted probabilities toward a specific class.

### Compared to other RPI predicting methods

In this work, we also compared LPI-MFF with current RPI prediction algorithms using the RPI1807 and NPInter datasets. RPITER, IPMiner, EDLMFC, and lncPro [50] were the most prevalent RPI prediction methods currently available for comparison. Table 6 displays the performance of different RPI prediction models on RPI1807 and NPInter as measured by seven evaluation indicators (ACC, SEN, SPE, PPV, F1, MCC, and AUC).

Table 6 reveals that in the RPI1807 dataset, RPITER had the highest SEN value of 97.94%, EDLMFC had the highest F1 value of 95.59%, and LPI-MFF had the highest ACC, SPE, PRE, MCC, and AUC values of 97.60%, 99.49%, 99.47%, 0.9755, and 99.57%, respectively. Additionally, RPITER had the highest SEN value in the NPInter dataset at 98.02%, IPMiner had the highest PRE and F1 values at 95.66% and 95.89%, and LPI-MFF had the highest ACC, SPE, MCC, and AUC values at 97.67%, 94.83%, 99.47%, and 0.9192, respectively. Moreover, both RPITER and EDLMFC had one evaluation index that is the highest in the RPI1807 data set, however, LPI-MFF had five evaluation indices that are the highest, accordingly, the LPI-MFF method should be used. Similarly, in the NPInter dataset, RPITER had one evaluation index that was the highest, IPMiner had two evaluation indices that were the highest, while LPI-MFF had four evaluation indices that were the highest, therefore, the RPI prediction effect using the LPI-MFF method was deemed superior. Thus, LPI-MFF is a good candidate for LPI prediction since it achieves superior prediction results on both datasets.

**Table 6:** Performance of LPI-MFF and other previous RPI prediction methods on RPI1807 and NPInter

Dataset	Method	ACC (%)	SEN (%)	SPE (%)	PPV (%)	F1 (%)	MCC	AUC (%)
	RPITER	96.87	**97.94**	95.54	96.50	95.31	0.9369	99.29
	IPMiner	96.80	96.51	97.82	95.56	94.87	0.9350	96.61
RPI1807	EDLMFC	93.35	96.62	83.71	94.60	**95.59**	0.8225	96.89
	lncPro	47.34	44.51	50.62	53.24	51.23	− 0.049	50.64
	LPI-MFF	**97.60**	95.72	**99.49**	**99.47**	95.27	**0.9755**	**99.57**
	RPITER	95.35	**98.02**	92.67	93.05	94.01	0.9083	98.56
	IPMiner	95.70	95.64	94.77	**95.66**	**95.89**	0.9140	95.77
NPInter	EDLMFC	96.14	97.19	92.13	93.63	94.35	0.9135	98.59
	lncPro	50.84	73.92	27.60	50.56	48.76	0.0170	51.72
	LPI-MFF	**97.67**	97.58	**94.83**	93.35	94.41	**0.9192**	**98.81**

Bold values represent the maximum value of the corresponding evaluation indicator

### Prediction of the independent dataset and Mus musculus RPI network

As evident from Table 7, in the RPI1168 dataset, RPITER had the highest SPE and F1 values, with respective values of 92.15% and 90.56%, IPMiner had the highest MCC value, reaching 0.7915, and LPI-MFF had the highest ACC, SEN, PRE, and AUC values, with respective values of 84.96%, 92.10%, 79.51%, and 88.97%. In the RPI1168 dataset, RPITER had two of the highest evaluation indicators, IPMiner had one of the highest evaluation indices, and LPI-MFF had four of the highest evaluation indicators, therefore, LPI-MFF was deemed the most effective predictor of RPI. Thus, LPI-MFF is a good candidate for LPI prediction because it achieves superior prediction results on RPI1168 and has high generalization ability.

**Table 7:** Performance of LPI-MFF and other previous RPI prediction methods on RP1168

Dataset	Method	ACC (%)	SEN (%)	SPE (%)	PPV (%)	F1 (%)	MCC	AUC (%)
	RPITER	69.82	54.81	**92.15**	70.10	**90.56**	0.4451	57.14
	IPMiner	77.43	73.55	88.17	69.28	56.14	**0.7915**	77.20
RPI1168	EDLMFC	81.46	87.85	76.14	72.39	84.15	0.6894	85.27
	lncPro	61.20	51.14	69.25	63.97	50.67	0.4115	46.12
	LPI-MFF	**84.96**	**92.10**	77.24	**79.51**	85.97	0.7214	**88.97**

Bold values represent the maximum value of the corresponding evaluation indicator

**Fig. 7:**
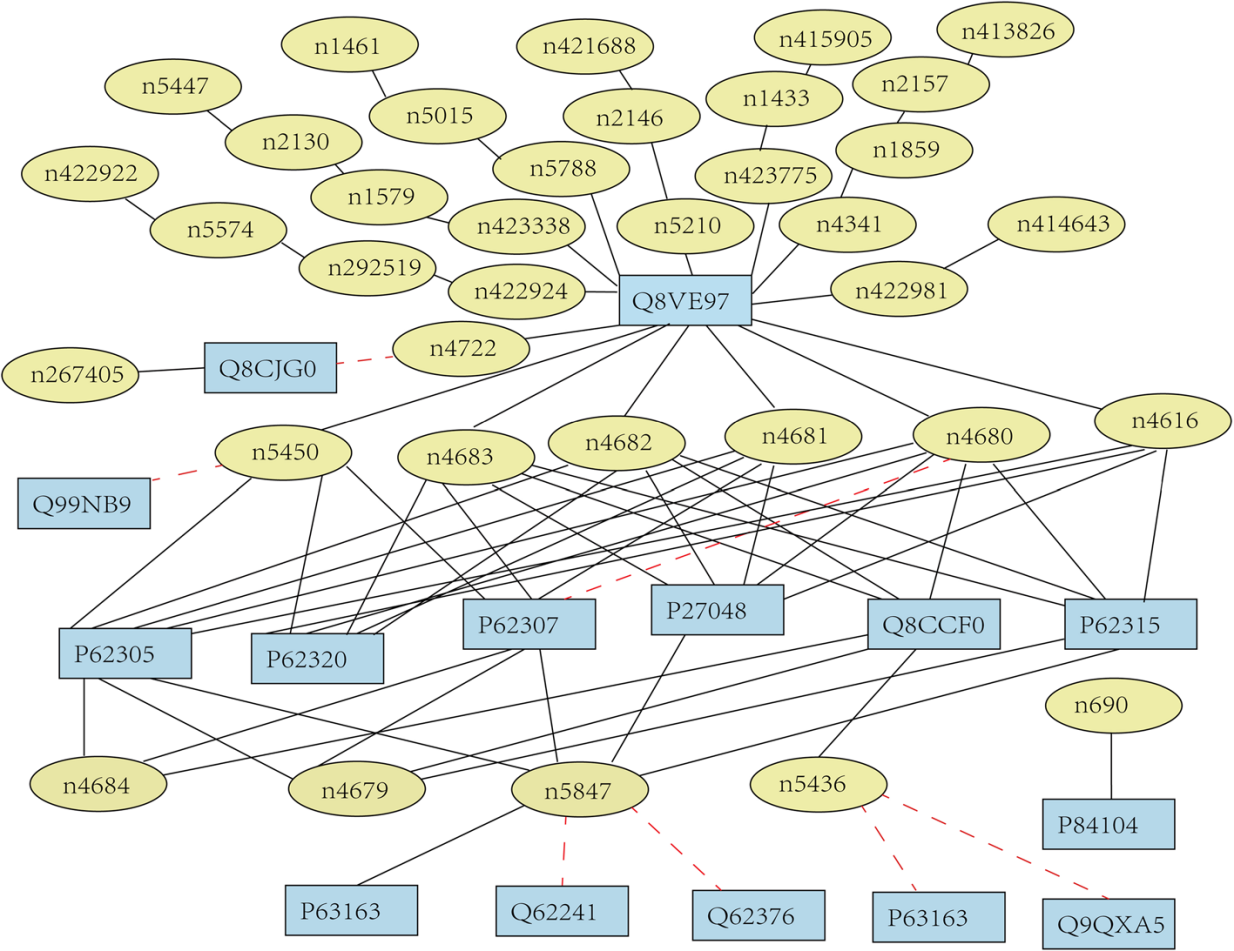
Prediction of RPI in Mus musculus dataset by LPI-MFF

In order to further test the generalization of LPI-MFF, we selected 77 Mus musculus RPI pairs from the NPInter v3.0 database [51], including 15 proteins and 36 lncRNAs, and tested the Mus musculus dataset using the LPI-MFF trained by RPI1807. As illustrated in Fig. 7, the ellipse represents lncRNA, the rectangle represents protein, the solid black line represents the accurate prediction of RPI by LPI-MFF, and the dashed red line represents the inaccurate prediction of RPI by LPI-MFF. As depicted, there were 70 black solid lines and 7 red dotted lines in the lncRNA–protein network of this study. This suggested that LPI-MFF correctly identified 70 pairs of RPIs and incorrectly predicted 7 pairs of RPIs, for a prediction accuracy of 90.91%, indicating that LPI-MFF’s prediction of the Mus musculus RPI network is still good. Additionally, the prediction of the RPI network enabled us to comprehend the role of RNA and protein in biological processes and conduct more in-depth research on the process of life processes [52], which was advantageous for drug discovery and cancer research. In this study, the protein Q8VE97, which interacted with the greatest number of lncRNAs in the lncRNA–protein network, inhibited the splicing of MAPT/Tau exon 10 by regulating the selection of alternative splicing sites during pre-mRNA splicing [53]. The protein P84104, which interacted with a single lncRNA, n690, is a splicing factor that promoted exon inclusion during alternative splicing [54]. Consequently, the prediction of these RPIs by LPI-MFF contributed to the study of alternative splicing and site replacement mechanisms. Furthermore, by making predictions within the lncRNA–protein network, we can gain a deeper understanding of the biological processes and functions of RNA-binding proteins.

## Discussion

Previous RPI prediction results were obtained through complex mathematical calculations. Consequently, the field of RPI forecasting necessitates a faster solution. In recent years, numerous RPI prediction calculation methods utilizing machine learning or deep learning have been introduced. These methods expedite the calculation process and reduce associated costs. This study therefore proposes LPI-MFF, a prediction method for the RPI based on deep learning. In this study, a comparative experiment was conducted to determine the effect of 11 feature combinations on the predictive performance of the model. Ultimately, PPI features, sequence features, secondary structure features, and physicochemical properties were used to predict. Use improved IK to extract sequence information of lncRNA, ICT to extract sequence information of protein, Fourier transform to extract secondary structure information of lncRNA and protein, the String database mapping method to obtain PPI information, and PC-PseAAC and DACC in the Pse-in-One tool to extract the physicochemical properties information of lncRNA and protein, respectively. Appropriate feature coding strategies are used to fully express distinct feature information, thereby increasing the evaluation index of model prediction RPI. Comparing the impact of various feature selection strategies on the accuracy of predictions led us to conclude that RF is the most effective feature selection strategy for this research model. Concurrently, we also conducted comparison experiments on the effect of the feature selection ratio of the feature vector on the prediction results, and we concluded that the model has the best predictive effect when the feature selection ratio of all feature vectors is 80%. In order to maximize feature vector utilization and improve calculation performance, we perform feature fusion on the four types of feature vectors present in this research. In this study, a parallel architectural model with four characteristics is designed. The four feature vectors are fused via the convolution layer, activation function layer, and pooling layer, respectively, prior to being fed to the softmax activation function via the fully connected layer for binary classification. On the basis of studies comparing LPI-MFF to other RPI prediction methods, we conclude that LPI-MFF has superior performance and the majority of assessment indicators are superior to those of existing RPI prediction methods. Despite the fact that this study has yielded relatively satisfactory results, there are still some shortcomings. For instance, the data set used for comparative experiments is comparatively small, the proportion of feature selection is not more specific, and the network structure is still overly complex.

## Conclusion

RPI prediction is essential to the study of physiological processes and the function of RNA and proteins in vivo. There have been numerous RPI prediction methods based on deep learning or machine learning in recent years. This study developed a deep learning-based LPI-MFF model for predicting RPI. First, four types of feature information, including PPI features, sequence features, secondary structure characteristics, and physicochemical attributes, are compiled. Second, encode the pertinent RNA feature information or protein feature information using mapping, ICT, DACC, PC-PseAAC, and Fourier transform, six feature encoding methods. Use RF as the feature selection algorithm of LPI-MFF, use RF to execute feature selection based on the Gini index, use the Gini index to calculate VIM, and choose the feature vector with the highest VIM to obtain the optimal feature vector. Utilize the concatenate feature fusion algorithm to perform feature fusion on the four feature vectors, maximize the use of feature vectors, and improve computational efficiency, and use the softmax activation function for binary classification. In this study, the accuracy of the LPI-MFF model was 97.60%, 97.67%, and 84.96% for RPI1807, NPInter, and RPI1168 respectively, which were all superior to other methods. The lncRNA–protein network achieved an accuracy of 90.91%, which is also quite good. Overall, LPI-MFF is a superior RPI prediction method. However, our model still has inherent limitations that require ongoing improvement for more precise predictions. Given the opportunity, we will expand the RPI dataset and curate higher-quality RPI and non-RPI pairs. Additionally, future comparative experiments will involve a larger set of feature selection ratios, as the current number is relatively small. Moreover, the network structure of our model is intricate and distinctive. To enhance the accuracy of our RPI prediction model, we plan to reconfigure the network topology, eliminate redundant modules, and integrate a state-of-the-art deep learning technique.

### Additional file

**Additional file 1**. Hyperparameter and sequence feature encoding pseudocode.

## Data Availability

The data and code are available at https://github.com/YingLiangjxau/LPI-MFF.
