# Supplementary material for: Predicting lncRNA–protein interactions through deep learning framework employing multiple features and random forest algorithm
Source: BMC Bioinformatics. 2024 Mar 12;25:108. doi: 10.1186/s12859-024-05727-4 (PMC10929084; doi:10.1186/s12859-024-05727-4)
Supplement: Supplementary file 1 — Additional file 1. Hyperparameter and sequence feature encoding pseudocode. [file 12859_2024_5727_MOESM1_ESM.docx]

**Table S1** The hyperparameters of random forest.

| Hyperparameters | Value |
| --- | --- |
| n_estimators (number of decision trees) | 500 |
| criteria (measurement method to evaluate the quality of feature segmentation) | "gini" |
| max_depth (maximum depth of decision trees) | 5 |
| max_features (number of features used for splitting at each node) | "auto" (all features) |

**Table S2** The hyperparameters of LPI-MFF.

| Hyperparameters | Value |
| --- | --- |
| Dropoute | 0.2 |
| Use_skip_connections | True |
| Activation function | RelU |
| Learning rate | 0.001 |
| Batch sizec | 32 |
| Optimizer | Adam |
| Loss function | Categorical cross entropy |
| Weight initialization | Kaiming |

The pseudocode for improved K-mer is as follows:

| Input: RNA sequence file;  Output: Feature vector after K-mer encoding of RNA sequence information; |
| --- |
| 1: Initialize sequence information, replace 'T' in the sequence with 'U'  2:Extract characters that match the defined elements from the sequence and concatenate them into a new string  3: Calculate sequence length  4: Define the variable result that stores the encoding result and the variable offset that records the offset of the encoding vector  5: Traverse K values from 1 to 4  6: Initialize a zero vector of length 4^k^  7: Calculate the number of cycles based on the K value and traverse each K-mer of the sequence  8: Extract K-mer  9: Update the count of the corresponding K-mer position in the vector  10: Convert vector to array and update offset  11: Normalize the vector  12: Convert the vector to a list and add it to the result  13: Convert the result to an array and return |

The pseudocode for improved conjoint triads is as follows:

| Input: protein sequence file;  Output: Feature vector after K-mer encoding of protein sequence information; |
| --- |
| 1: Initialization sequence information  2:Extract characters that match the defined elements from the sequence and concatenate them into a new string  3: Calculate sequence length  4: Define the variable result that stores the encoding result and the variable offset that records the offset of the encoding vector  5: Traverse K values from 1 to 3  6: Initialize a zero vector with length 7^k^  7: Calculate the number of cycles based on the K value and traverse each K-mer of the sequence  8: Extract K-mer  9: Update the count of the corresponding K-mer position in the vector  10: Convert vector to array and update offset  11: Normalize the vector  12: Convert the vector to a list and add it to the result  13: Convert the result to an array and return |
